# The learning curve of single-port laparoscopic appendectomy performed by emergent operation

**DOI:** 10.1186/s13017-016-0096-z

**Published:** 2016-08-05

**Authors:** YongHun Kim, WooSurng Lee

**Affiliations:** 1 Department of Surgery, School of Medicine, Konkuk University, Konkuk University Chungju Hospital, 82, Gugwon-daero, Chungju-si, Chungbuk Republic of Korea; 2 Department of Thoracic and Cardiovascular Surgery, School of Medicine, Konkuk University, Konkuk University Chungju Hospital, 82, Gugwon-daero, Chungju-si, Chungbuk Republic of Korea

**Keywords:** Learning curve, Laparoscopy, Appendectomy, Emergencies

## Abstract

**Background:**

Single-port laparoscopic appendectomy (SPLA) has the advantage of minimizing abdominal incision scars with patient satisfaction. However, it has the following disadvantages: it provides a narrower surgical field than conventional laparoscopic appendectomy, which requires a considerably longer operative time to achieve surgical skills. This study was conducted to evaluate the learning curve for SPLA.

**Methods:**

This study included a total of 120 patients with acute abdomen who visited our emergency department and were diagnosed with acute appendicitis between March 2013 and February 2015. They underwent SPLA by a single surgeon. Patients were divided into 4 groups of 30 patients each according to operation dates. Operative time, time to resume oral intake, length of hospital stay, and postoperative complications were analyzed.

**Results:**

The mean operative time was 59.9 ± 19.9 min. It was shortened after completion of 30 operations and remained unchanged until it was further shortened after completion of 90 operations. There was no significant difference in time to resumption of oral intake or length of hospital stay between the 4 groups. Postoperative complications occurred in 18 patients, but the frequency of the complications was not significantly different between the 4 groups.

**Conclusions:**

The results of this study suggest that surgeons can achieve surgical skills for SPLA after completion of 30 operations and more experienced surgical skills by SPLA successfully after completion of 90 operations.

## Background

Laparoscopic appendectomy is minimally invasive compared to conventional open appendectomy and has the advantages of decreased postoperative pain, shortened hospital stay, fewer postoperative complications, and better cosmesis. It has replaced conventional open appendectomy in the treatment of acute appendicitis [1, 2]. Therefore, this laparoscopic appendectomy is currently the gold standard operation for acute appendicitis and provides similar surgical outcomes as conventional open appendectomy, even in cases of complicated appendicitis [3]. Recently, advances in laparoscopic instruments and optical systems enabled surgeons to perform intra-abdominal operation through a single incision around the umbilicus; in particular, single-port laparoscopic appendectomy (SPLA), which minimizes visible scars in the abdominal wall, satisfies patients [4, 5]. In addition, previous randomized controlled studies reported that SPLA has surgical outcomes similar to those of three-port laparoscopic appendectomy (TPLA) [6, 7]. However, since SPLA has the disadvantages of limited surgical field and difficult access to the operation site through a small incision compared to TPLA, it requires more experience with surgical cases and more skills than TPLA, especially in emergency cases. For this reason, to achieve surgical outcomes similar to those of TPLA, SPLA demands additional training programs. Recent preliminary studies have not yet completely elucidate the learning curve of SPLA [8, 9]. Thus, the aims of this study were to analyze the learning curve of SPLA over a longer period and to assess its surgical safety and feasibility.

## Methods

This study included a total of 122 patients with acute abdomen who visited our emergency department and were diagnosed with acute appendicitis between March 2013 and February 2015. They underwent SPLA. To make a definite diagnosis of acute appendicitis, all patients were evaluated using a comprehensive history taking, physical examination, laboratory findings, and abdominal computed tomography with contrast enhancement. Each patient gave informed consent to SPLA. Two patients had undergone right hemicolectomy for peritonitis with severe cecal inflammation and diverticular perforation, who were excluded from the study. All operations were performed by a single surgeon in the same surgical team who had experience with more than 500 TPLA cases and more than 500 conventional open appendectomy cases. Patients were consecutively assigned to 4 groups of 30 patients each: group A (the first 30 patients), group B (the second 30 patients), group C (the third 30 patients), and group D (the fourth 30 patients). Clinical data on age, gender, body mass index (BMI), severity of appendicitis, and previous history of abdominal surgery was collected and analyzed with regard to operative time, postoperative complications, length of hospital stay, time to resume oral intake, and conversion rate.

### Surgical procedure

**Fig. 1:**
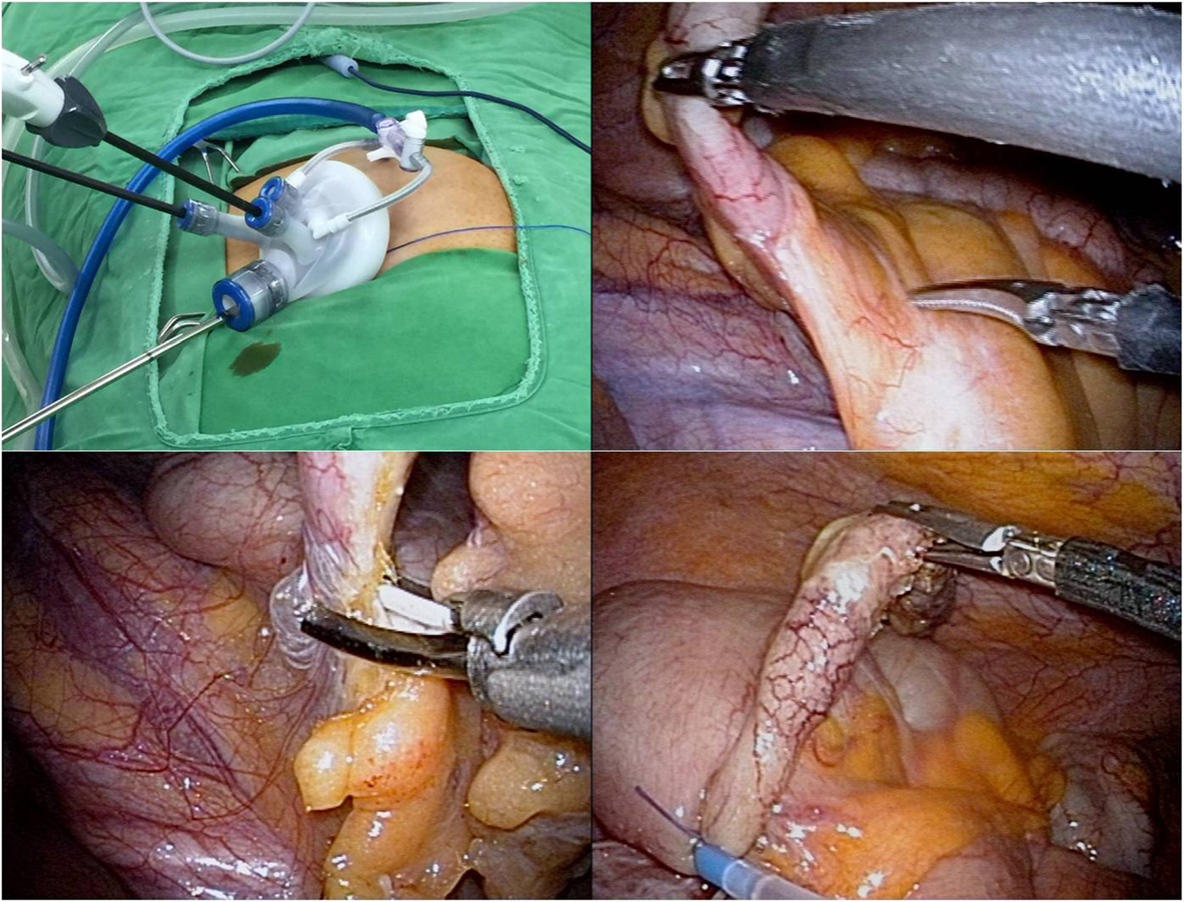
Surgical procedure of single-port laparoscopic appendectomy

SPLA was performed under general anesthesia. The patent lay on the operating table in the supine position, with both arms lying outside. Both the surgeon and assistant stood on the patient’s left side. After a 2-cm mid-line linear incision was made just above the umbilicus, the abdominal wall was opened using the Hassan technique. Once proper umbilical access has been obtained, a Glove Port (Nelis, Seoul, Korea) was positioned and secured within the incision, providing both 360° wound protection and circumferential atraumatic retraction. Pneumoperitoneum was maintained at 10–12 mm Hg using CO_2_. SPLA was conducted using a 5-mm Flexible EndoEYE laparoscope (Olympus Surgical & Industrial America Inc, Center Valley, PA, USA) and a 5-mm HiQ™ Curved Instrument (Olympus Surgical & Industrial America Inc, Center Valley, PA, USA). The appendix was grasped and pulled with a 5-mm HiQ™ Curved Instrument immediately on its tip, if visible, or starting from its base or proximal third and gradually mobilizing the entire body of the appendix. When the appendix was stuck by surrounding dense and thick inflammatory adhesions, the use of a blunt suction device could be extremely safe and useful for creating a plane for dissection and mobilization from the surrounding inflamed viscera and tissues. Once the appendix was fully mobilized and its base on the cecum was identified, the mesentery was carefully dissected and coagulated using a Harmonic scalpel (Ethicon, Blue Ash, OH, USA). Once the base of the appendix on the cecum was reached and the mesenteric tissues were fully cleansed, the appendix base was ready to be knotted using a Surgitie™ loop (Covidien, Mansfield, MA, USA). Finally, after cutting the appendix, the specimen was retrieved under direct vision and extracted through the umbilical glove port. The operation was terminated with aspiration and eventual cautious lavage, especially in the right iliac fossa and Douglas pouch, checking hemostasis on the mesentery and good closure of the appendiceal stump (Fig. 1).

### Statistical analysis

Data analysis was performed using MedCalc for Windows version 12.3.0 (MedCalc Software, Ostend, Belgium). In statistical testing, a two-sided *P* value of >0.05 was considered statistically significant. Continuous variables are expressed as mean ± standard deviation. Categorical variables are presented as frequency and percentage. In univariate analysis, Fisher’s exact test and one-way analysis of variance were used as appropriate for group comparisons. Then, multivariate analysis was conducted by fitting multiple linear regression models to identify important predictors of operative time.

## Results

**Fig. 2:**
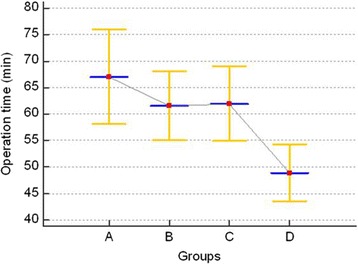
Changes in operative time according to operation dates

The mean age of the patients was 45.1 ± 16.1 years (range, 19.0–87.0 years), and there were 67 males and 53 females. The mean BMI was 23.38 ± 3.20 (range, 17.0–34.8). Of the 120 patients, 16 had a history of abdominal surgery: 1 had cholecystectomy, 9 had cesarean section, 3 had myomectomy, and 3 had tubal ligation. There was no significant difference in age, gender, BMI, or history of abdominal surgery between the 4 groups (Table 1). The types of appendicitis classified according to severity were as follows: the suppurative (*n* = 83), gangrenous (*n* = 24), and perforated (*n* = 13) types. The appendices were categorized according to their location as follows: the retrocecal (*n* = 18), subcecal/pelvic (*n* = 76), and retroileal (*n* = 26) types. The mean operation time was 59.9 ± 19.9 min, and there was no intra-abdominal injury or massive bleeding during operation. Of the 120 patients, 4 required additional port insertion, all of whom belonged to group A. There were no patients who required conversion to open appendectomy. The mean time to resume oral intake was 1.5 ± 0.7 days, but it was not significantly different between the 4 groups (*P* = 0.418) Postoperative complications occurred in 18 patients, and most of them were wound complications (*n* = 11, 9.1 %). There were four patients (3.3 %) with intra-abdominal inflammation which was resolved by antibiotics therapy, without percutaneous drainage. Postoperative ileus occurred in three patients (2.5 %), which was improved by conservative treatment. There was no significant difference in postoperative complications between the 4 groups (*p* = 0.853) (Table 1). Mean operative times were 67.0 ± 23.9 min in group A, 61.7 ± 23.9 min in group B, 60.4 ± 17.5 min in group C, and 50.6 ± 16.8 min in group D. Mean operative times were longest in group A and shortest in group D. One-way analysis of variance (ANOVA) showed significant correlations between groups A and D, and between groups B and C (*P* = 0.012). The mean operative time was shortened after completion of 30 operations and remained unchanged until it was further shortened after completion of 90 operations (Fig. 2). In multiple linear regression analysis, the severity of appendicitis was significantly associated with operative time; however, history of abdominal surgery, location of the appendix, or BMI was not significantly associated with operative time (Table 2).

**Table 1:** Patients demographics and comparisons of learning curve-related variables

	Overall	Group A	Group B	Group C	Group D	*P value*
Age (years)	45.1 ± 16.1	43.8 ± 17.3	44.9 ± 10.8	44.6 ± 15.2	47.1 ± 17.8	0.297
Gender (M/F)	67/53	14/16	14/16	20/10	19/11	0.245
BMI (kg/m^2^)	23.38 ± 3.2	23.58 ± 3.3	23.02 ± 3.4	23.63 ± 3.1	23.30 ± 2.9	0.874
Previous history of AS	16	5	4	3	4	0.901
Operation time (minutes)	59.9 ± 19.9	67.0 ± 23.9	61.7 ± 17.9	60.4 ± 17.5	50.6 ± 16.8	0.012
Additional port insertion	4	4	0	0	0	0.006
Time to ROI (days)	1.5 ± 0.7	1.5 ± 0.8	1.6 ± 0.8	1.5 ± 0.6	1.6 ± 0.8	0.966
Hospitalization (days)	5.1 ± 2.5	5.3 ± 2.7	5.2 ± 2.3	4.9 ± 1.9	4.8 ± 2.0	0.418
Complications	18	5	5	5	3	0.853
wound problem	11	3	3	4	1	0.230
intraabdominal infection	4	1	1	1	1	1.000
ileus	3	1	1	0	1	0.890

*Abbreviations*: *M/F* male/female, *BMI* body mass index, *AS* abdominal surgery, *ROI* resumption of oral intake

**Table 2:** Multivariate analysis of predictors of operative time

	Coefficient	Standard error	*T* value	*P* value
Group	-5.26	1.52	-3.45	0.0008
Severity of appendicitis	7.95	2.53	3.14	0.0021
BMI	1.14	1.78	0.63	0.524
Location of appendix	-0.89	2.75	-0.32	0.746
Past history of abdominal surgery	-2.99	5.01	-0.59	0.552

By the multiple linear regression model: *n* = 120, residual standard error = 18.6, *R* ^2^ = 0.126, overall F test statistic = 4.448, *P* = 0.001 (*df* = 5, 114)

*Abbreviation*: *BMI* body mass index

## Discussion

Single-puncture tubal ligation via a laparoscopic single-opening approach was first introduced by Wheeless in 1969 [10]. Thereafter, this approach has been employed in the treatment of complicated gynecological diseases and applied for appendectomy, placement of peritoneal dialysis catheters, and resection of intra-abdominal cysts [11, 12]. Despite such applications in various clinical conditions, single-port laparoscopic surgery (SPLS) has not yet been widely performed in real-world practice. The reasons for this may be the limitations of SPLS to clinical indications and the steep learning curve to overcome. A major problem is difficulty in manipulating the laparoscope and the instruments introduced through a single port because surgeons should avoid extra- and intracorporeal conflict between the laparoscope and the instruments. Such drawbacks frequently lead to the loss of triangulation and difficult instrumentation during SPLS, unlike conventional multiport laparoscopic surgery. SPLS requires the surgeon and the assistant to maintain a poor ergonomic position different from that in conventional laparoscopic surgery. SPLS may be more difficult to perform than conventional laparoscopic surgery, prolong operative time, require a steep learning curve, and have low feasibility and safety [11–13]. For addressing these issues, many improved devices have been used in real-world practice: longer laparoscopes (45 cm in length), laparoscopes with various angles, improved port systems, such as Home-made Glove port, SILS™ Port (Covidien Inc, Norwalk, CT, USA), Uni-X™, AirSeal (SurgiQuest, Orange, CT, USA), Glove port (Nelis, Seoul, South Korea), and laparoscopic instruments specifically designed for SPLS, such as Roticulator™ (Covidien Inc, Norwalk, CT, USA), HiQ LS™ Curved instrument (Olympus Surgical & Industrial America Inc, Center Valley, PA, USA) [11, 12]. Despite such advances in laparoscopes and instruments, surgeons should have a substantial learning curve to perform SPLS.

Recently, SPLA has been extensively performed because of the superior cosmesis with no visible scarring [14]. Most of the previous studies have reported that the clinical outcomes of SPLA are the same as those of TPLA [4–7]. Meanwhile, some studies have reported that SPLA requires a longer operative time and a substantial learning curve [15]. Liao et al*.* [8] analyzed 30 cases of non-complicated appendicitis undergoing SPLA and documented that the operative time for SPLA was shortened after experience with 10 cases of SPLA, which became the same as that for TPLA. Validad et al*.* [9] investigated 65 pediatric cases of acute appendicitis undergoing SPLA and showed that the operative time for SPLA was equal to that for TPLA, regardless of board certification. These studies may have some limitations to interpret their results in that they excluded cases of complicated appendicitis. However, our study analyzed 120 cases of acute appendicitis which included cases of complicated appendicitis, so that it has clinical implications in such aspects. Moreover, since our study included only SPLA cases during the study period, with exclusion of TPLA cases, we avoided selection bias and obtained more general data on the learning curve for SPLA. The learning curve for SPLA can be assessed by various parameters, such as blood loss, complication rates, conversion rates, operative time, and length of hospital stay, of which operative time and length of hospital stay are more important [16, 17]. In this study, the operative time for SPLA was shortened after completion of 30 SPLA cases, remained unchanged, and was further shortened significantly after completion of 90 SPLA cases. Since previous studies on the learning curve for SPLA had a relatively small sample size (*n* = 20–50), they may have some limitations to generalize their results [16–19]. Pan et al*.* [20] divided 180 consecutive single-incision laparoscopic cholecystectomies into 9 groups according to operation dates, and each group included 20 patients operated on consecutively in each time period. They also reported that the operative time was significantly longer in group 1 than in the other groups. Lee et al*.* [21] divided 160 cases of myoma uteri requiring single-port laparoscopic myomectomy into 4 groups according to operation dates and reported that the operative time declined significantly in the last 3 groups compared to the first group. Our results on the progressive decrease in the operative time for SPLA can provide useful information on a general learning curve for SPLA. Further studies are needed to confirm our results.

In this study, the learning curve for SPLA showed no significant changes in the length of hospital stay between the 4 groups, which is similar to the results of previous studies [8, 22, 23] Additionally, there was no significant difference in the occurrence of postoperative complications between the 4 groups, which is similar to the results of previous studies [8, 23]. In TPLA, the complication rates have been shown to decreases to <10 % with the mastery of the TPLA technique [18, 24]. In this study, the complication rate of SPLA turned out to be 15 %, mainly including wound complications such as seroma (*n* = 9), which may have attributed to longer umbilical incisions and a larger proportion of cases of complicated appendicitis. The complication rates of SPLA was acceptable with the exclusion of seroma (9/120, 7.5 %).

**Fig. 3:**
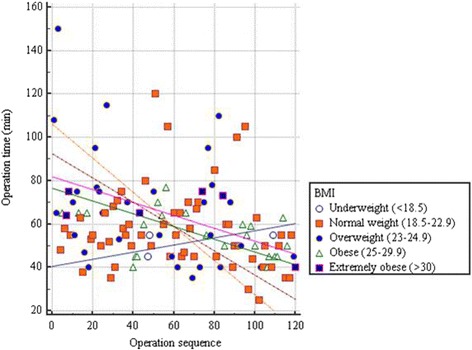
Correlation of operation time and BMI

There have been only a few studies on clinical predictors associated with the operative time for SPLA. Liao et al*.* [8] have documented that operative time for SPLA is related to the severity of appendicitis, BMI > 21 kg/m^2^, and use of endoloops. Lee et al*.* [24] have shown that the operative time for SPLA is not associated with leukocyte count or duration of symptoms; however, it is associated with BMI and pathology of the appendix. In this study, the operative time for SPLA was associated with the severity of appendicitis, but not with BMI or the location of the appendix. However, BMI seems more likely to be a significant factor that can affect the operation time. In our study, it was found that there was a less reduction in the operative time in the obese and extremely obese patients compared to normal weight patients, but it was not so in underweight patients (Fig. 3), suggesting that BMI may not be significantly associated with operative time. Based on these results, it is conceivable that the severity of appendicitis may affect the operative time for SPLA. Another considerable limitation of SPLA is related to costs. The increased costs of SPLA compared to TPLA are still a considerable disadvantage that limits the use of SPLA [5, 25]. Di Saverio et al*.* [26] analyzed 45 cases of single-incision laparoscopic appendectomy using a self-made surgical-glove port and showed that the postoperative results from their surgical method were comparable to those from conventional single-incision laparoscopic appendectomy. They suggested that their surgical-glove port, single-incision laparoscopic appendectomy could be considered as a cost-effective alternative to conventional SPLA using commercially available devices.

This study has a limitation because all the SPLA cases were performed by a single surgeon in the same surgical team. Further studies are needed to achieve a general learning curve for SPLA.

## Conclusions

The results of this study suggest that equipment conflict, difficulty in manipulating the laparoscope and the laparoscopic instruments through a small umbilical incision, and limited surgical field may hamper the clinical application of SPLA, but that the surgical outcome can be the same as that of TPLA. Even surgeons proficient in TPLA will need a substantial learning curve to safely perform the SPLA technique.

## Abbreviations

BMI, body mass index; SPLA, single-port laparoscopic appendectomy; SPLS, single-port laparoscopic surgery; TPLA, three-port laparoscopic appendectomy.
